# Extramedullary Hematopoiesis of the Urinary Bladder Associated With Metastatic Prostatic Adenocarcinoma: A Case Report

**DOI:** 10.7759/cureus.93516

**Published:** 2025-09-29

**Authors:** Jirawat Chuaykaew

**Affiliations:** 1 Anatomical Pathology, Chonburi Hospital, Chonburi, THA

**Keywords:** extramedullary hematopoiesis, hematuria, hemorrhagic cystitis, primary myelofibrosis, prostatic neoplasms, urinary bladder

## Abstract

Extramedullary hematopoiesis (EMH) is the production of hematopoietic cells outside the bone marrow, which most commonly occurs in the liver, spleen, and lymph nodes. EMH involving the urinary bladder is exceptionally rare, with only a few cases reported. We present the first known case of bladder EMH secondary to bone marrow metastasis from prostatic adenocarcinoma. The patient presented with gross hematuria; cystoscopy revealed multiple bladder masses. Histopathological examination demonstrated erythroid and myeloid precursors, along with scattered megakaryocytes, confirmed by immunohistochemistry. Bladder EMH should be considered in the differential diagnosis of patients with solid tumors presenting with hematuria. Hemorrhagic cystitis can mimic this entity and lead to misdiagnosis. The awareness of this rare manifestation is essential for accurate diagnosis and appropriate management.

## Introduction

The formation of hematopoietic elements outside the bone marrow is referred to as extramedullary hematopoiesis (EMH). It is a compensatory mechanism to maintain physiological homeostasis and is usually associated with myeloproliferative neoplasms [1]. Patients with EMH may be asymptomatic or present with symptoms related to the site of involvement, such as organomegaly, bleeding, mass-like lesions, or pain. The most common sites of EMH include the liver, spleen, and lymph nodes. EMH involving the urinary bladder is extremely rare, with only a few cases reported [2-4].

In advanced malignancy, the disruption of marrow hematopoiesis can precipitate EMH [5]. Notably, among solid tumors, prostate cancer shows the highest prevalence of bone metastases (88.74%), a pattern that is clinically relevant because bone involvement and marrow dysfunction may promote extramedullary hematopoietic responses [6]. Consistent with this concept, Romero-Laorden et al. described a patient with prostatic adenocarcinoma who developed hepatic EMH in the absence of detectable metastatic disease, concluding that advanced prostate cancer can itself be a primary cause of EMH [7]. Building on this observation, we describe a distinct anatomic presentation: urinary bladder EMH occurring in the setting of metastatic prostatic adenocarcinoma. This case expands the spectrum of prostate cancer-associated EMH and highlights a rare, clinically relevant mimic of bladder neoplasia; to our knowledge, such a presentation has not previously been reported.

This case report was granted an exemption by the Institutional Review Board (IRB) of Chonburi Hospital.

## Case presentation

A 67-year-old Thai man presented with abdominal discomfort, back pain, and unexpected weight loss. He had no known underlying medical conditions and denied any history of trauma. A complete blood count revealed a decreased hematocrit of 24% (normal range: 40%-54%), a reduced platelet of 43,000/µL (normal range: 150,000-450,000/µL), and the presence of nucleated red blood cells (Table 1). These findings led to a diagnosis of bicytopenia (anemia and thrombocytopenia).

**Table 1:** Laboratory tests MCV, mean corpuscular volume; MCH, mean corpuscular hemoglobin; NRBC, nucleated red blood cells

Laboratory test	Observed value	Reference range	Unit
White blood cells (WBC)	4.99	4.0-11.0	10^3^/µL
Red blood cells	2.92	4.5-6.0	10^6^/µL
Hemoglobin	8.1	14-18	g/dL
Hematocrit	24	40-54	%
MCV	82.2	83-97	fL
MCH	27.7	27-33	pg
Platelet count	43	150-450	10^3^/µL
Platelet smear	Decrease	Adequate	-
NRBC	1	-	cells/100 WBC

**Figure 1:**
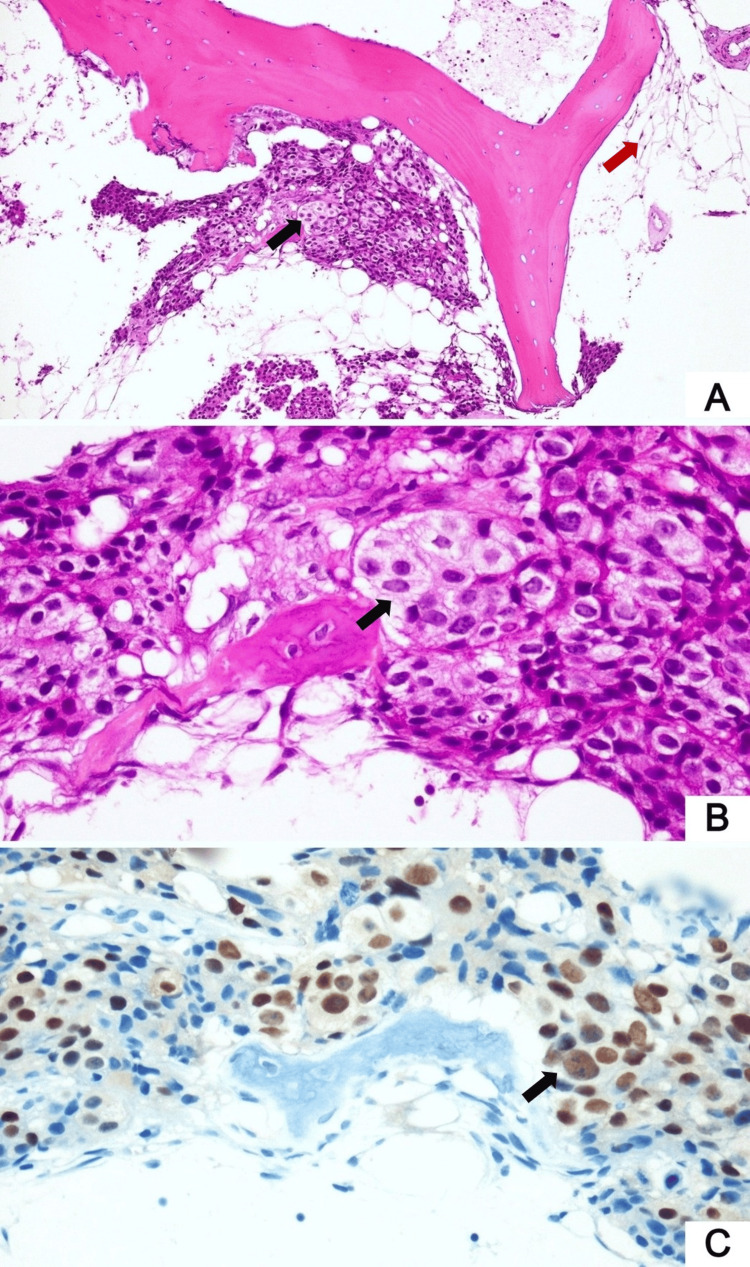
A bone marrow biopsy reveals hypocellular marrow replaced by fatty tissue (red arrow) (A, ×100) with foci of metastatic prostatic adenocarcinoma (black arrow) (A, ×100; B, ×400), which demonstrates positive staining for NKX3.1 (C, ×400) Hematoxylin and eosin

Despite this, he reported no signs or symptoms of bleeding. A bone marrow aspiration and biopsy were performed to evaluate the underlying cause, revealing a hypocellular marrow infiltrated by a metastatic prostatic adenocarcinoma, confirmed by NKX3.1 immunopositivity (Figure 1).

**Figure 2:**
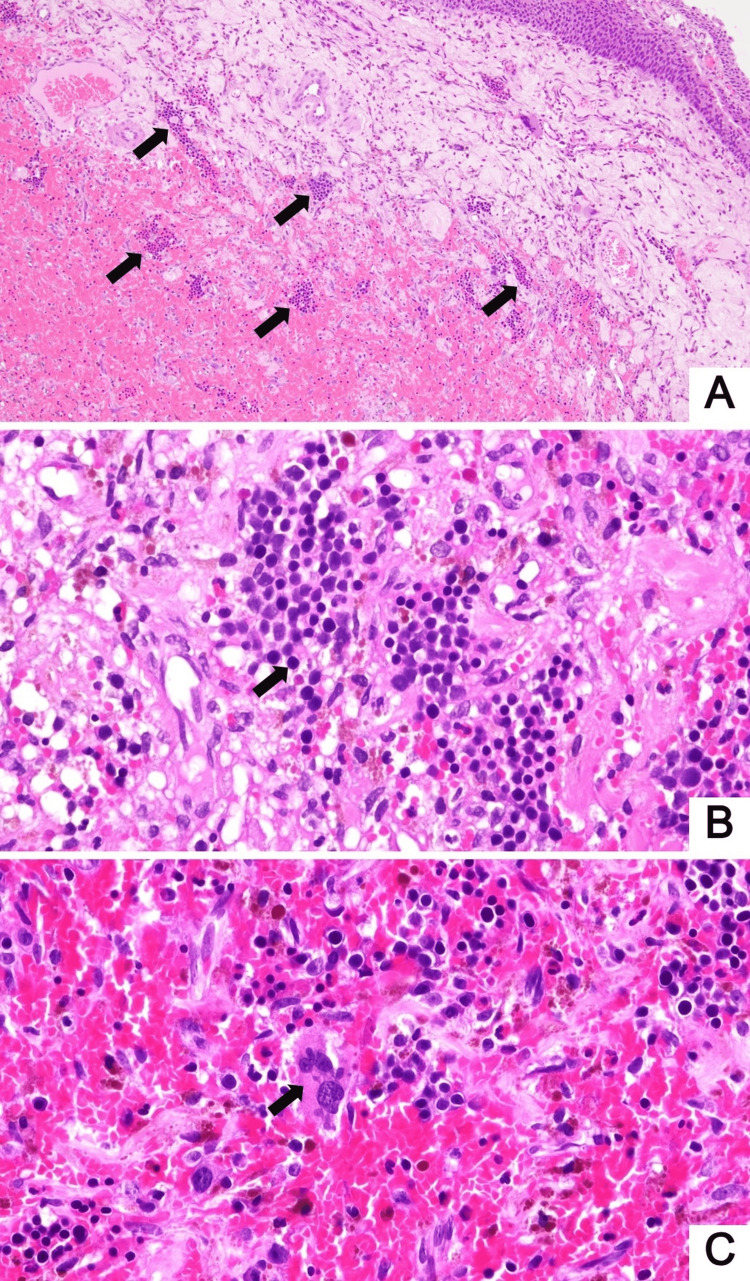
Histologic features of extramedullary hematopoiesis in the urinary bladder reveal small clusters of myeloid and erythroid precursors (black arrow) (A, ×40). At higher magnification, myeloid and erythroid precursors are observed (B, ×400). A hyperlobated megakaryocyte is also present (arrow) (C, ×400) Hematoxylin and eosin

Five days later, the patient developed gross hematuria. Cystoscopy revealed multiple sessile bladder masses ranging from 2 to 3 cm in diameter, some partially covered with blood clots. A transurethral resection of bladder tumor (TUR-BT) was performed to rule out bladder metastasis. Histologic examination of the bladder masses demonstrated scattered small clusters of round blue cells within a hemorrhagic and edematous lamina propria. The overlying urothelium was unremarkable (Figure 2A). Occasional large cells with hyperlobated nuclei and abundant cytoplasm were also observed (Figure 2C).

**Figure 3:**
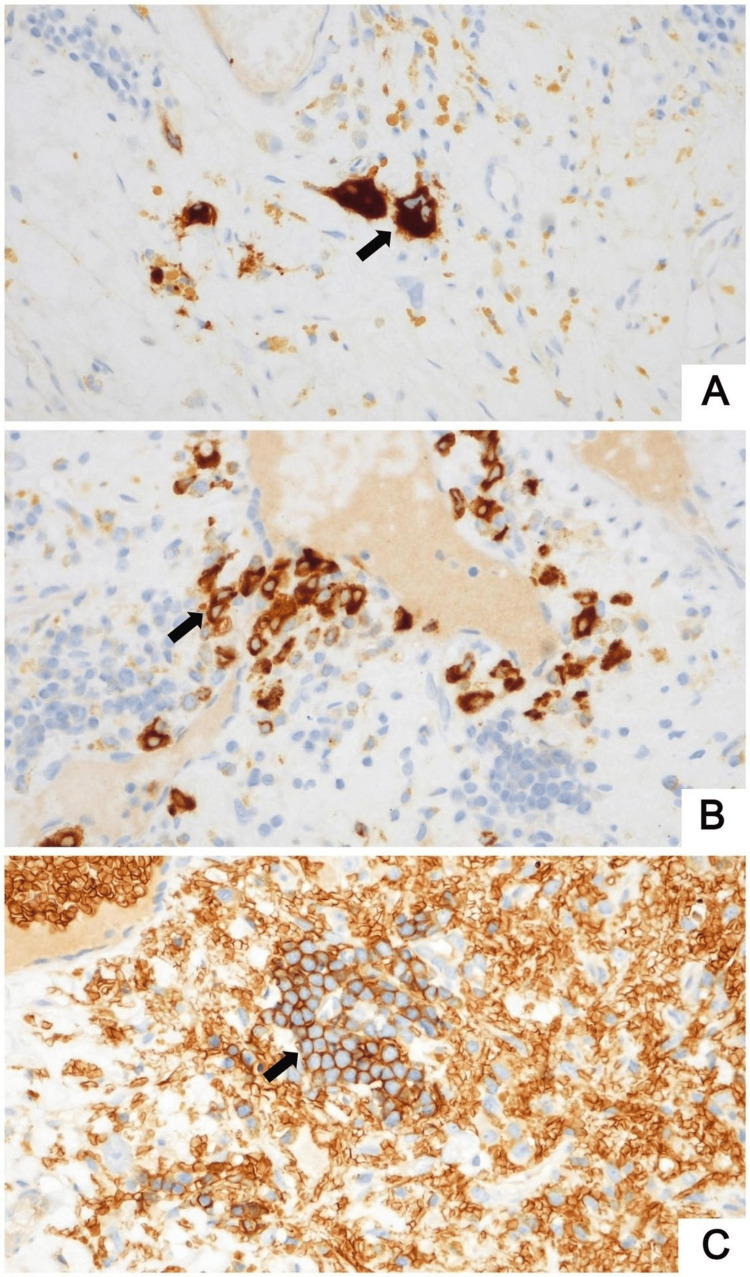
Immunohistochemical studies demonstrate CD61-positive megakaryocytes (A, ×400), MPO-positive myeloid precursors (B, ×400), and glycophorin C–positive erythroid precursors (C, ×400) MPO, myeloperoxidase; CD, cluster of differentiation

Immunohistochemical staining for pancytokeratin (clone AE1/AE3) was negative, excluding metastatic prostatic adenocarcinoma. Cluster of differentiation 34 (CD34) and terminal deoxynucleotidyl transferase (TdT) were also negative, ruling out leukemic infiltration. Interestingly, myeloperoxidase (MPO), CD61, and glycophorin C (Figure 3) were positive, highlighting myeloid precursors, megakaryocytes, and erythroid precursors, respectively. These findings were diagnostic of EMH.

**Figure 4:**
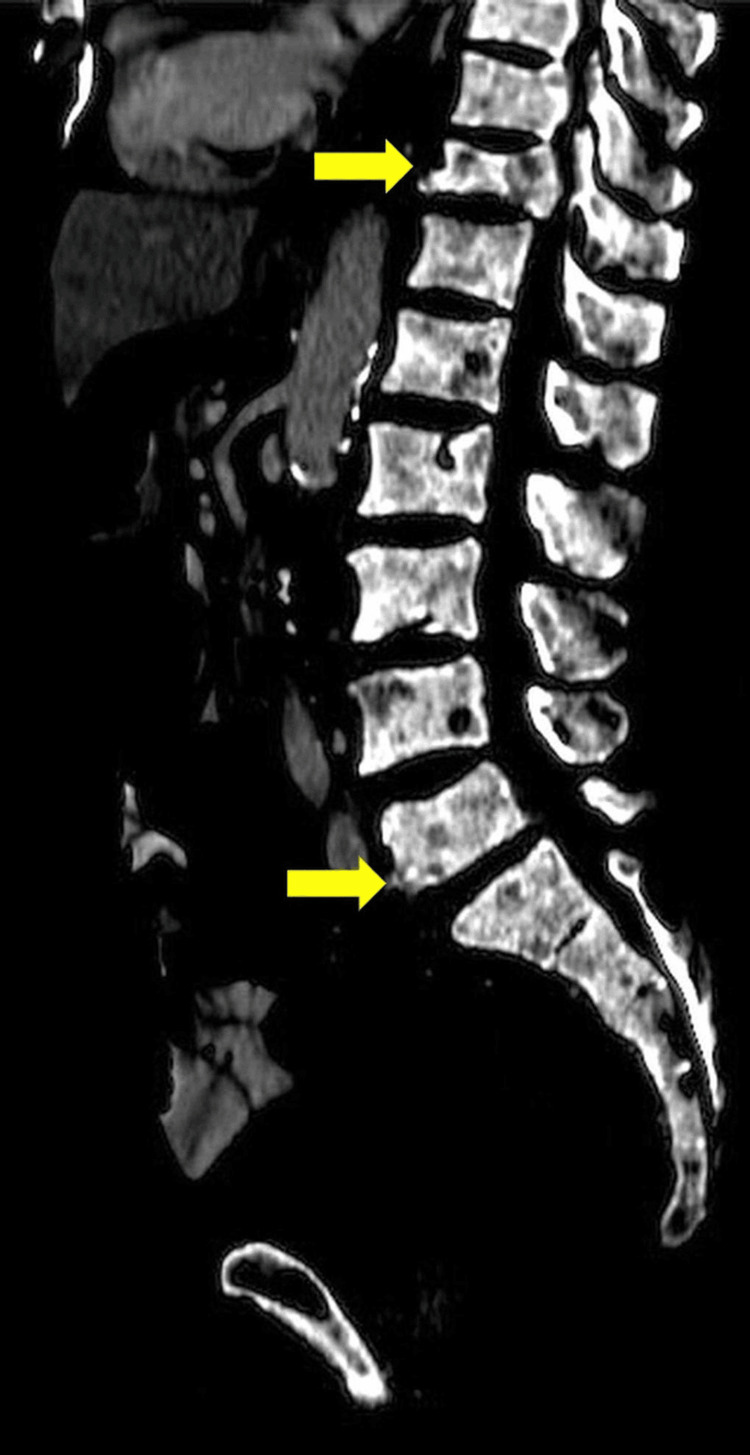
A sagittal CT scan of the whole abdomen demonstrated diffuse mixed osteoblastic and lytic lesions involving multiple bony structures, along with the collapse of the T11 vertebra (upper yellow arrow) and the L5 vertebra (lower yellow arrow) CT: computed tomography

Following TUR-BT, there was no recurrence of gross hematuria. A subsequent transurethral resection of the prostate (TUR-P) was performed, confirming the diagnosis of prostatic adenocarcinoma with a Gleason score of 9. The computed tomography (CT) scan of the whole abdomen revealed diffuse mixed osteoblastic and lytic lesions involving multiple bony structures, along with the collapse of the T11 and L5 vertebrae, consistent with extensive bone metastases (Figure 4). The patient underwent bilateral orchiectomy as part of his treatment for metastatic prostate cancer. Unfortunately, he passed away four months later due to pulmonary embolism and septicemia.

## Discussion

Extramedullary hematopoiesis (EMH) refers to hematopoietic activity occurring outside of the bone marrow. It can be categorized into active and passive forms [8]. During embryonal development, the yolk sac is the initial site of hematopoiesis, followed by the liver and spleen [9]. From approximately the third trimester of gestation through adult life, the bone marrow becomes the primary site of hematopoietic cell formation [10]. These processes are referred to as active EMH. In contrast, passive EMH occurs when the bone marrow fails to maintain adequate hematopoiesis, as seen in conditions such as infection, metabolic stress, anemia, and cancer [11]. EMH is most commonly associated with myeloproliferative neoplasms, especially primary myelofibrosis, with frequent involvement of the spleen, liver, and lymph nodes [1]. Less common sites such as the lung, appendix, and kidney have been reported [12-14]. Patients with EMH may be asymptomatic or present with symptoms related to the site of involvement, such as organomegaly, bleeding, mass-like lesions, or pain. As previously mentioned, the EMH of the urinary bladder is exceedingly rare. To date, only three cases of bladder EMH have been reported in the English-language literature, all associated with idiopathic myelofibrosis and presenting with gross hematuria similar to our case [2-4]. To our knowledge, this is the first reported case of bladder EMH associated with a malignant solid tumor, which is prostatic adenocarcinoma with bone marrow metastasis.

EMH in the context of solid tumors is rare. Bao et al. found that EMH was most frequently seen in patients with breast cancer, most often involving lymph nodes [5]. Several mechanisms have been proposed to explain EMH in solid tumors. Chen et al. reported that tumor-derived cytokines such as granulocyte-macrophage colony-stimulating factor (GM-CSF), tumor necrosis factor-alpha (TNF-α), and interleukin-6 (IL-6) can suppress marrow erythropoiesis and induce erythroid apoptosis, thereby facilitating EMH [11]. Bao et al. stated that extensive bone marrow metastasis disrupts the marrow microenvironment, promoting EMH [5]. Moreover, chemotherapy such as doxorubicin has also been implicated as a potential trigger of EMH in animal models [15,16]. Beyond its associated clinical symptoms, EMH may also directly influence tumor behavior. Some studies have reported that erythroid progenitor cells (EPCs) derived from tumor-induced EMH suppress CD8+ T-cell function, thereby impairing antitumor immunity and the efficacy of immunotherapy and promoting tumor growth [11,17,18]. In this case, our patient had not received any prior treatment. Based on the CT report, we hypothesize that widespread metastases in our case may have contributed to the development of EMH.

Currently, there is no standardized treatment guideline for EMH. Management is individualized based on the location and clinical context. For example, blood transfusion is recommended for patients with thalassemia to help reduce the size of mass lesions. Surgical resection may be considered in cases where the lesion is accessible and operable. On the other hand, for unresectable lesions, radiation therapy has been utilized as an alternative treatment option [19].

The pathologic diagnosis of EMH at uncommon sites can be challenging due to overlapping histologic features with other conditions, depending on the affected organ. Wang and Darvishian have stated that the EMH of the breast may mimic invasive lobular carcinoma [16]. For our case, metastatic prostatic adenocarcinoma with a single-cell pattern (i.e., Gleason pattern 5) may closely resemble EMH. However, the absence of cytokeratin (AE1/AE3) immunoreactivity helps exclude occult prostatic adenocarcinoma and supports a non-epithelial origin of the lesion.

Another condition that may mimic EMH, due to its shared hematopoietic lineage, is leukemic infiltration, particularly acute megakaryoblastic leukemia (AMKL). Both entities are composed of loosely cohesive small round cells and megakaryocytes, and both are CD61-positive. Nevertheless, this case is not consistent with AMKL for two main reasons. First, AMKL predominantly occurs in pediatric patients rather than adults. Second, AMKL typically presents as a hypercellular lesion characterized by the marked proliferation of megakaryoblasts and megakaryocytes. In contrast, this case exhibits lower cellularity and contains only a small number of megakaryocytes.

Finally, hemorrhagic cystitis is an important differential diagnosis and diagnostic pitfall in cases of bladder EMH. It is a more commonly encountered condition, and immunohistochemical studies are not typically required for its diagnosis. Moreover, it shares overlapping histologic features with EMH, such as hemorrhagic lamina propria and the presence of erythroid precursors that may resemble small lymphocytes. However, hemorrhagic cystitis typically demonstrates ulceration with a combination of acute and chronic inflammation, and the presence of large hyperlobated cells is not characteristic. Therefore, we propose that the identification of large hyperlobated cells, which are characteristic features of megakaryocytes, should raise suspicion for EMH and prompt confirmatory immunohistochemical staining.

## Conclusions

Although the EMH of the urinary bladder is exceedingly rare, recognizing this entity, particularly in patients with solid tumors presenting with gross hematuria, can facilitate accurate diagnosis and avoid unnecessary interventions. The presence of multilobated megakaryocytes serves as a useful histopathological clue in differentiating EMH from its mimickers. While no standardized treatment for EMH currently exists, surgical resection may aid in symptom control, as demonstrated in our case.
